# Psychometric evaluation of the student nurse stressor-14 scale for undergraduate nursing interns

**DOI:** 10.1186/s12912-023-01631-z

**Published:** 2023-12-08

**Authors:** Jingjing Ding, Yang Yu, Jie Kong, Qing Chen, Patricia McAleer

**Affiliations:** 1 The First Affiliated Hospital of Jinzhou Medical University, Jinzhou, China; 2 Jinzhou Medical University, Jinzhou, China; 3 NetwellCASALA, Dundalk Institute of Technology, Dundalk, Co., Louth, Ireland

**Keywords:** Undergraduate nursing students, Clinical practice, Stress, Reliability, Validity

## Abstract

**Background:**

Currently, there are few scales used to assess the stressors experienced by undergraduate nursing interns during clinical practice, and the assessment of stressors during clinical practice is not comprehensive; the scale includes some unique stressors during training that is not available in the existing instruments used to assess nursing student practice stress in China.

**Aim:**

The study aimed to explore the structure of the Chinese revision of the Student Nurse Stressor-14 Scale(SNS-14-CHI)and investigate the psychometric properties it among Chinese undergraduate nursing interns.

**Methods:**

The original scale was culturally adjusted and revised after expert correspondence on the entries, and 414 undergraduate nursing interns were recruited from three cities in China to administer the questionnaire. Reliability was measured by internal consistency, fold-half reliability, and stability. Content validity was evaluated using exploratory factor analysis (EFA) and confirmatory factor analysis (CFA) to assess the validity of the SNS-14-CHI.

**Results:**

The SNS-14-CHI retained 14 items, the EFA supported a 2-factor structure, and the items’ factor attribution differed from the original scale. The CFA results showed a good model fit. The Cronbach coefficient of the scale was 0.934, and the coefficient values of the two factors were 0.890 and 0.898. The content validity index of the scale was 0.964.The cumulative variance contribution of the 2-factor structure was 60.445%. The split-half reliability and stability were 0.869,0.762, respectively.

**Conclusion:**

The SNS-14-CHI has excellent reliability and validity among undergraduate nursing trainees. The evaluation results of the scale can provide a reference for nursing managers to develop educational programs and interventions to quantify nursing student stress.

**Supplementary Information:**

The online version contains supplementary material available at 10.1186/s12912-023-01631-z.

## Introduction

Stress has been described as a non-specific response of individuals when faced with negative things or circumstances [1, 2]. Studies have shown that stress generated in the environment can exacerbate biological vulnerability [3]. The clinical learning environment is a significant source of stress for nursing students [4]. Stress is an aggravating or triggering factor that threatens health [5]. It is also widely recognized as a psychosocial factor that hinders nursing students’ academic and clinical practice [6–9]. Nursing students report more frequent levels of stress than students in other professional degree programs [7], and they typically experience moderate to high-stress levels [10]. Although nursing students are not subject to the same responsibilities as formally registered nurses during clinical practice, they are sometimes often exposed to the same stressors; unlike other professions, nursing students are required to take some responsibility for the health of their patients, which means saying goodbye to their student campus life and possibly sacrificing some of their social time with their peers in a usual way [11]. Studies have summarized the main stressors faced by nursing students: academic stressors related to clinical training or clinical stressors (patient care, relationships with clinical staff, lack of professional knowledge and skills, practical tasks and workload); and external stressors (financial burdens, personal or social stressors), most of which occur during clinical placements [12–14].

Nursing, as an applied science, focuses on integrating theory and practice. In China and other countries, completion of a practical learning phase involving direct patient care is necessary to qualify for a nursing degree [15, 16]. Clinical practice is part of nursing education and a critical period for nursing students to enhance their competencies and skills. Studies have shown that clinical practice contributes to the psychological development of nursing students and improves social skills and adaptation to professional roles [17]. The experience gained in real clinical situations during clinical practice helps nursing students to understand nursing expertise and recognize the true nature of the nursing profession, as well as to enhance nursing students’ knowledge and skills in the nursing field [18]. Although clinical placements are significant for the development of the nursing discipline and the nursing students themselves, the stress students experience during clinical practice should not be overlooked. Students with higher stress levels are prone to errors in clinical work, which can seriously threaten patient safety [19]. In addition, stress during clinical placements can lead to physical and psychological symptoms, such as headaches, anxiety, stress, sleep deprivation, attention deficit, cognitive decline, and learning difficulties, which can reduce the quality of nursing and even shake their belief in pursuing a career in nursing [20]. Therefore, exploring the stress profile and stressors of nursing interns during the clinical placement phase helps them improve their coping skills and thus reduce the adverse effects of stress.

Currently, the scale used to assess stress among nursing students in China is the Nursing Student Stress Index Scale [21], validated among undergraduate nursing students with good reliability and validity. Still, the instrument is not specific for assessing stressors during nursing practice. Based on the literature review and quantitative research, Irish academic Patricia Sheridan developed The Student Nurse Stressor-15 Scale (SNS-15) in 2019 for use and validation in undergraduate geriatric nursing interns (17–25 years old), which was primarily used to assess undergraduate geriatric nursing interns’ Sources of stress during the internship, the scale consists of 15 items, two dimensions resources, knowledge and workload [22], the scale is currently not validated for use in other countries.

Compared to other instruments measuring nursing student stress, this instrument quantifies unique stressors in the clinical setting, such as days of missing attendance, length of journey for placement, days worked per week, facilities, and so on. According to the 2008 CNA Regulations, nursing students are required to participate in 8 months of clinical practice, and lack of days of attendance can affect eligibility for registration to practice as a participating nurse. The travel distance of the placement is also stressful for the students as they need to take transportation to the clinical site. Suppose the operating hours of public transit do not match the working hours. In that case, most students will not be able to take transportation, so they will choose to pick a place to stay near the internship hospital, which will bring additional accommodation costs and financially burden them. The number of days per week is a source of stress for internship students, some of whom will be assigned to the same shift work pattern as regular nurses, with less free time. Lack of adequate facilities for clinical placements may reduce motivation to pursue a career in nursing. There is a lack of research instruments to effectively assess the above stressors in China. This study aimed to translate the SNS-15 into Chinese, adapt it to the Chinese cultural context, and validate its reliability among undergraduate nursing interns.

## Aims and expected results

Due to the different cultural backgrounds of China and Ireland and the different measurement populations, there should be some differences in the content aspects of the scales. In this study, we assessed the psychometric properties of the original scale among Chinese undergraduate nursing interns. We hypothesized that the Chinese scale version had good reliability and validity.

## Instruments and methods

### Translation and culture adaptation

Before translating and validating the Student Nurse Stress Scale, we obtained permission from the original scale authors to process the scale strictly following the Brislin two-person translation-back-translation model [23]. (1) Translation: Two English graduate students translated the English version of the scale into Chinese separately and formed the first draft of the Chinese version of scale A after careful discussion and revision with the researcher. (2) Back translation: 2 English experts who had not been exposed to the original scale were invited to independently back-translate the first draft of the Chinese version of scale A into English. (3) Cross-cultural debugging: First, to adapt the assessment tool to Chinese expression habits, the meaning behind each item was confirmed with the original author by email. Second, one psychologist and nursing expert carefully discussed and compared the original scale, the first draft A of the Chinese translation, and the back-translated scale. Controversial items were modified to align the scale content with Chinese reality. The original scale had 15 items and two factors. For item 8, which deals with the ability to access resources, these resources mainly include wheelchairs, bed sheets, toiletries, or personal care products, as the direct contacts of this equipment and items in China are patients’ families and caregivers, and nursing interns have little contact with them, this item does not apply to the clinical internship scenario in Chinese hospitals. So, experts suggest deleting item 8 and forming a Chinese version of SNS-14-CHI. A pre-survey was conducted with ten undergraduate nursing interns to find out how the students understood the content of the scale and how they felt when completing the scale.(for the final English version of the SNS-14-CHI, see supplementary document).

### Design and study population

This study was conducted from December 2022 to March 2023, using a convenience sampling method to select undergraduate nursing interns from internships in Shenyang, Dalian, and Jinzhou, Liaoning Province, China, as respondents. To ensure the accuracy of the exploratory factor analysis(EFA) and confirmatory factor analysis (CFA), there were at least ten survey participants per item [24]. Participants should meet the following criteria : (1) full-time undergraduate nursing interns; (2) informed consent and voluntary participation in this study. Interns who were not on duty due to medical leave were excluded. The researcher personally contacted the person in charge of each internship base and obtained the consent of the students to distribute the questionnaires; all questionnaires were collected on-site, 420 questionnaires were distributed, 6 were excluded due to incomplete completion, and 414 valid questionnaires were collected, with a reasonable return rate of 98.6%. All survey participants’ information was anonymous except for the 30 participants selected, who were asked to write down their student number and contact information. 2 weeks later, the 30 participants who were numbered and left their contact information were tested to retest the reliability. The investigator provided informed consent before all survey participants.

### Instruments

The questionnaire consists of three parts: demographic variables, SNS-14-CHI and PSS-14.

General Demographic Characteristics Questionnaire: This questionnaire includes six self-report items. Age, gender,  experience of student leaders, whether they like their major, homeplace, and household income status.

The Student Nurse Stressor-14 Scale: the Student Nurse Stressor-15 Scale for nursing students developed by Patricia Sheridan [22] measured the stress levels of Irish geriatric nursing trainees and the questionnaire consisted of 15 items with two dimensions: the knowledge and workload domain and the domain of the resource. A Likert scale was used to measure from 1 to 5, corresponding to (1) Highly stressed, (2) Stressed, (3) Neutral, (4) Moderately stressed, and (5) Not stressed. A lower score rarely indicates a higher level of stress. One item was selected (removed from the Chinese version), and higher scale scores indicated that nursing interns felt a lower stress level. The translation of the Student Nurse Stress15 scale into the revised Chinese Student Nurse Stressor-14 Scale (SNS-14-CHI) has been discussed previously.

The Perceived Stress Scale (PSS-14) is a standardized test instrument. This study used a revised version by Chinese scholar Yang Yanzhong to assess participants’ stressful situations, showing good reliability and Validity in China [25]. This measure consists of 2 dimensions with 14 items: a sense of loss of control (items 4, 5, 6, 7, 9, 10, and 13), which is reverse scored, and tension (items 1, 2, 3, 8, 11, 12, and 14). The scale was scored on a 5-point Likert scale, with higher scores associated with tremendous stress.

## Data analysis

SPSS26.0 and AMOS24.0 software was used to analyze the data. Mean ± standard deviation (x ± s) was used to describe the quantitative data; frequency and composition ratio were used to describe the qualitative data.

### Validity analysis

EFA and CFA were used to explore and validate the potential factor structure of SNS-14-CHI. 414 undergraduate nursing interns were randomly divided into two groups: EFA (n = 207) and CFA (n = 207). The scale is suitable for factor analysis only when the KMO > 0.6 and the Bartlett spherical test is statistically significant (*P* < 0.05) and combined with a visual inspection of lithographs for factor extraction. Amos 24.0 was used to test the factor model in CFA.

### Item analysis

The powerful value method was applied to evaluate the discrimination of the items. The total scores of the SNS-14-CHI were ranked from high to low, the first 27% were taken as the high group, and the last 27% as the low group, and the independent samples t-test was used to compare the differences in the mean values of the item scores. The correlation of each item of the translation scale with the total score, combined with the deleted Cronbach’s alpha coefficient, was used to assess whether each item of the translation scale was retained.

### Content validity

Six experts were invited to evaluate the content validity of the SNS-14-CHI using the Delphi method. The content validity of the SNS-14-CHI was independently assessed on a 4-point scale of “not relevant” (1 point), “weakly relevant” (2 points), “strongly relevant” (3 points), and “strongly relevant” (4 points). “The I-CVI is the ratio of the number of experts who ranked each item with a score of 3 or 4 to the total number of experts, and the S-CVI is the average of the I-CVI of all items.

### Criterion validity

In this study, the PSS-14 was used as a criteria tool to make preliminary inferences about the Validity of the SNS-14-CHI.

### Reliability analysis

The coefficient was used to test the internal consistency of the SNS-14-CHI by dividing the post-test translation questions into two halves, calculating the correlation coefficient between the two halves of the test, and using this as an estimate of the folded reliability of the test. 2 weeks later, the retest reliability was tested on the 30 undergraduate nursing interns who were flagged.

## Results

### Descripitive statistics

A total of 414 undergraduate nursing interns were included in this study, 117 (28.3%) males and 297 (71.7%) females; Age ranged from 20 to 27, with a mean value of 22.77 ± 1.685; the number of rural and urban origin of birth was 226 (54.6%) and 188 (45.4%), and the rest is shown in (Table 1).

**Table 1:** Sample characteristics

Variables	Total(N = 414) N(%)/(Mean ± SD)	EFA(N = 207) N(%)/(Mean ± SD)	CFA(N = 207) N(%)/(Mean ± SD)
Age in years	22.77 ± 1.685	22.75 ± 1.632	22.78 ± 1.739
Gender			
Male	117(28.3)	67(32.4)	50(24.2)
Female	297(71.7)	140(67.6)	157(75.8)
Homeplace			
Rural areas	226(54.6)	116(56.0)	110(53.1)
Urban areas	188(45.4)	91(44.0)	97(46.9)
Household income status			
≥ 30,000RMB/per month	27(6.5)	16(7.7)	11(5.3)
≥ 10,000RMB/per month	151(36.5)	73(35.3)	78(37.7)
> 5000RMB/per month	212(51.2)	105(50.7)	107(51.7)
≤ 5000RMB/per month	24(5.8)	13(6.3)	11(5.3)
Experience of student leaders			
Yes	201(48.6)	99(47.8)	102(49.3)
No	213(51.4)	108(52.2)	105(50.7)
Like the nursing major or not			
Yes	320(77.3)	156(75.4)	164(79.2)
No	94(22.7)	51(24.6)	43(20.8)

### Item analyze

For the scale SNS-14-CHI, the decision values for each item ranged from 12.832 to 22.467 (*p* < 0.001), all of which were more significant than 3.0, and all 14 items were retained. The Pearson’s correlation coefficients between each item and the total score were 0.654 to 0.790 (*p* < 0.001), indicating that the scale items were highly correlated with the total score. After deleting each item, Cronbach’s α coefficients value of the SNS-14-CHI was 0.927 to 0.931. The scale’s internal consistency would not improve, indicating that all 14 items should be retained (Table 2).

**Table 2:** Item analysis for Chinese version of the Student Nurse Stressor-14 Scale

Item	Item score(SD)	Cronbach’s Alpha if item deleted	t-test	Correlation coefficient between item and total score
1	3.66(1.12)	0.931	−12.832	0.654
2	3.02(1.15)	0.931	−16.690	0.679
3	3.30(1.10)	0.928	−19.271	0.761
4	3.30(1.12)	0.928	−19.684	0.761
5	3.28(1.12)	0.927	−19.287	0.790
6	3.15(1.15)	0.928	−18.725	0.769
7	2.91(1.22)	0.931	−17.627	0.692
9	3.57(1.12)	0.929	−17.353	0.740
10	3.81(1.10)	0.930	−13.615	0.688
11	3.57(1.13)	0.929	−16.607	0.737
12	3.24(1.13)	0.927	−20.210	0.774
13	3.65(1.18)	0.927	−22.467	0.784
14	3.27(1.25)	0.929	−20.757	0.749
15	3.67(1.16)	0.930	−15.416	0.687

### Validity analysis

#### Construct validity

**Fig. 1:**
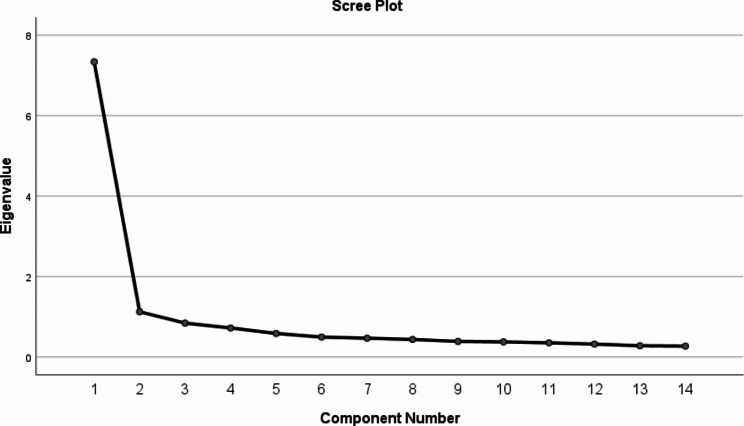
Screen plot of exploratory factor analysis of SNS-14-CHI

In the EFA, KMO = 0.941, and Bartlett’s spherical test was statistically significant (χ2 = 1582.967; *P* < 0.001), above the minimum value of 0.6, indicating a good fit for factor extraction. Principal component analysis extracted two factors with eigenvalues of > 1.00, accounting for 60.445% of the total variance. Table 3 shows the factor loadings for each item, where items 1, 14, and 15 entries were attributed differently from the original scale. The factor structure was confirmed among crushed stones, with a slow decreasing trend after 2 points (see Fig. 1).

**Table 3:** Factor loadings of exploratory factor analysis for the SNS-14-CHI

Items	Interpersonal Relationships and Resource	Knowledge and Workload
S11	0.780	
S10	0.779	
S9	0.769	
S13	0.613	
S14	0.609	
S1	0.574	
S15	0.528	
S12	0.527	
S2 S7		0.822 0.808
S6		0.742
S4		0.670
S5		0.628
S3		0.589

**Fig. 2:**
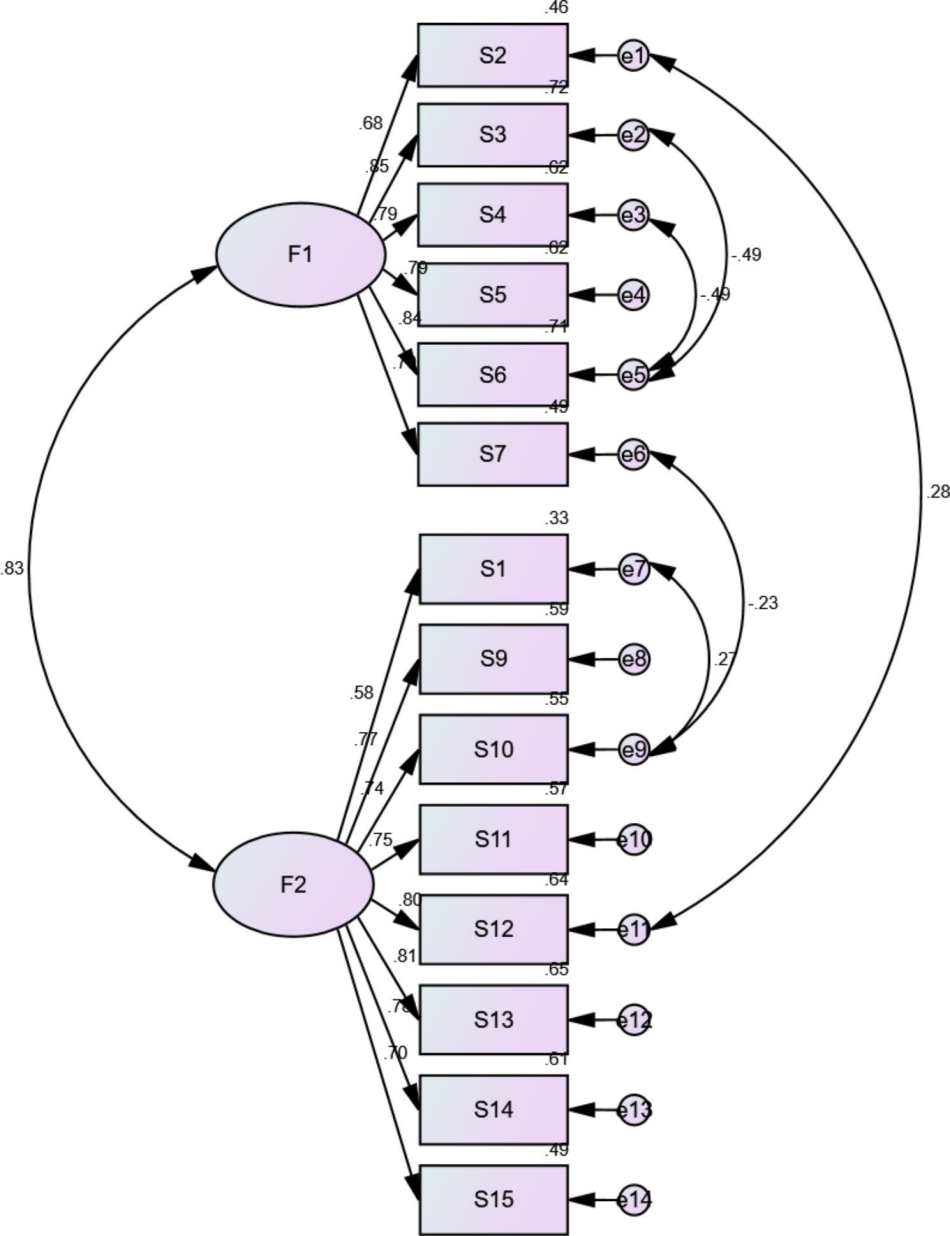
Standardized two-factor structural model of SNS-14-CHI (n = 207)

The CFA’s 2-factor model fit indices were within an acceptable range. CMIN/DF (chi-square/degree of freedom) = 2.160, GFI(goodness of fitness index) = 0.904, RMSEA (root mean square error of approximation) = 0.075, CFI (comparative fit index) = 0.955, NFI(normed fit index) = 0.920, TLI (Tucker Lewis index) = 0.942, and IFI (incremental fit index) = 0.955, the final model fitting indices are shown in (see Table 4 and Fig. 2).

**Table 4:** Result of the confirmatory factor analysis of SNS-14-CHI(n = 207)

Items	χ2/df	GFI	RMSEA	CFI	NFI	TLI	IFI
Fitting standards	≤ 3.00	> 0.09	< 0.08	> 0.09	> 0.09	> 0.09	> 0.09
Fitting results	2.160	0.904	0.075	0.955	0.920	0.942	0.955

#### Content validity

Six experts were invited to rate the content validity of SNS-14-CHI, with an I-CVI of 0.833 to 1.000 and an S-CVI/Ave of 0.964 (see Table 5).

**Table 5:** Item-level CVI for the SNS-14-CHI.

Items	Expert1	Expert2	Expert3	Expert4	Expert5	Expert6	I-CVI
1	4	2	4	4	4	4	0.833
2	4	4	4	3	4	4	1
3	4	3	4	4	4	3	1
4	4	4	4	3	3	4	1
5	4	4	3	4	4	4	1
6	4	4	4	4	4	4	1
7	3	4	4	3	4	4	1
9	4	3	4	3	3	3	1
10	4	4	3	3	4	4	1
11	4	4	4	4	3	4	1
12	4	3	4	3	4	4	1
13	3	4	2	3	3	3	0.833
14	4	4	4	2	3	4	0.833
15	4	4	3	3	4	4	1

#### Criterion validity

In this study, validity analysis was conducted using correlation analysis to make preliminary inferences about the Validity of the SNS-14-CHI. The universal scale was used as a validity tool in this study. Correlation analysis between the PSS-14 and the SNS-14-CHI scale showed a negative correlation and a statistically significant difference (r=-0.369, *p* < 0.001), indicating that the SNS-14-CHI can be used to assess stress among undergraduate nursing interns in a Chinese setting.

#### Reliability analysis

Cronbach’s total alpha for the SNS-14-CHI was 0.934. Cronbach’s alpha coefficients on each factor were 0.890 and 0.898. In addition, the fold-half reliability of the scale was 0.869. 2 weeks later, a random sample of 30 undergraduate nursing interns obtained a retest reliability of 0.762. (Table 6)

**Table 6:** Reliability analysis for Chinese version of the Student Nurse Stressor-14 Scale

The scale and its dimension	Cronbach’s Alpha	split-half reliability	Test-retest reliability
SNS−14-CHI	0.934	0.869	0.762
Knowledge and Workload	0.890		
Interpersonal Relationships and Resource	0.898		

### Differences in characteristics of stress among undergraduate nursing interns

The results of the variance analysis are shown in Table 7. The result of the analysis of differences showed that factors influencing the total score on the SNS-14-CHI included household income status and whether the nursing major or not.

**Table 7:** Comparison of SNS-CHI-14 scores for stress with different characteristics

	M	SD	t/F	P-value	Pairwise differences
Gender			1.232	0.219	
Male	48.51	12.536			
Female	46.93	11.432			
Homeplace			−1.123	0.262	
Rural areas	46.79	11.465			
Urban areas	48.09	12.099			
Household income status			12.407	**0.000**	(1)>(2)>(3),(4)
≥ 30,000RMB/per month	55.59	10.649			
≥ 10,000RMB/per month	50.01	11.680			
> 5000RMB/per month	45.12	11.406			
≤ 5000RMB/per month	41.58	8.097			
Experience of student leader			1.919	0.056	
Yes(1)	48.52	11.664			
No(2)	46.31	11.778			
Like the nursing major or not			3.945	**0.000**	(1)>(2)
Yes(1)	48.59	11.133			
No(2)	43.24	12.911			

## Discussion

In this study, we first underwent cultural adaptation by strictly following the Brislin double-translation model and expert opinions to form a revised Chinese version of the SNS-14 scale. We verified that the SNS-14-CHI has good reliability and validity and is particularly suitable for assessing stress in undergraduate nursing interns.

The content validity results of the SNS-14-CHI scale showed that the I-CVI ranged from 0.83 to 1.00, and the S-CVI/Ave was 0.964, which is higher than the normal reference values of 0.780 and 0.900 for content validity [26], suggesting better content validity of the scale. Our findings support a 2-factor structure consisting of 14 items compared to the original 15-item two-factor structure of the English scale. After expert deliberation, it was recommended that item 8 be removed from the original scale. The original scale: knowledge and workload (items 1, 2, 3, 4, 5, 6, 7, 14, 15) and resources (items 8,9,10,11,12,13) totaled 15 items. The study’s EFA revealed that the first factor had six items related to the original scales (2, 3, 4, 5, 6, and 7) and was named “knowledge and workload.“ Factor 2 had eight items, including the original scales (1, 9, 10, 11, 12, 13, 14, and 15). Combining the existing literature, expert opinion, and the potential characteristics of these items, we renamed it “Interpersonal relationships and resources.”

In this study, factor analysis was used to describe the structural validity of the SNS-14-CHI. EFA identified two factors in 14 items that explained 60.445% of the total variance. A factor loading of 0.60 or higher for each item was considered ideal [27].

However, the attribution of entries differed from the original scale. The researchers classified (entries 1, 14and 15) as Factor 2 and renamed them in conjunction with the references explained as follows: the noted relationship of Peplau’s interpersonal theory is essential in nursing practice [28]. Although Peplau’s interpersonal theory focuses on the nurse-patient relationship, the emphasis on partnership in nurse-patient interactions also applies to nursing students in clinical learning. According to Bandura, learning occurs in socialization [29], and positive relationships between clinical nursing teachers (lead teachers) and nursing students are what enhance clinical learning [28]. The role of the mentor in China differs from that of the mentor in Ireland, where the part of the mentor is that of a clinical registered nurse who provides clinical supervision and assessment of students and mentoring work in academic areas.

In contrast, the mentor role in China is mainly filled by university faculty, who are nursing educators who teach and mentor students in academic aspects, ideological guidance, professional counseling, life guidance, career guidance, and psychological guidance. Studies have shown that the interpersonal relationships that nursing educators build with students may positively correlate with students’ clinical adjustment [30]. Students feel that care from faculty inspires confidence, creates an atmosphere of learning and knowledge, and better demonstrates their professional autonomy; mentors set a good role model for students and positively promote good interpersonal relationships between students and patients [31]. Regardless of the country of study, interpersonal relationships between students and university faculty and with ward staff during clinical placements are essential for learning [32].

Studies have shown that staff absenteeism positively correlates with the distance from accommodation to work, with longer distances associated with higher staff absenteeism [33]. An online survey study of Australian university nursing students on placement found that most respondents faced financial difficulties during their clinical placements. The cost of transportation was identified as one of the most important factors [16]. From another perspective, it was explained that the placement distance is an essential resource for clinical placement students. If the distance from the hospital to the accommodation is closer, the smaller the transportation cost the student pays and the less financial burden. Therefore, factor 2 of the original scale (items 9, 10, 11, 12, and 13) was renamed from “resources” to “interpersonal relationships and resources” (items 1, 9, 10, 11, 12, 13, 14, and 15). We also believe the differences may be related to the different medical environments, cultural backgrounds, and disciplinary education in China and abroad. The various educational approaches may lead to a further understanding of the issues.

Applying CFA revealed that the model fit was statistically significant for CMIN/DF < 3, (GFI), TLI, CFI, NFI, GFI, and IFI > 0.9, (RMSEA) < 0.08 [34], and both methods indicated good structural Validity of the SNS-14-CHI.

There was a significant negative correlation between the SNS-14-CHI and the PSS-14 (r=-0.369, *p* < 0.001). Studies have shown that clinical learning environments are significantly correlated with students’ perceived stress levels [4]. The higher the score of SNS-14-CHI, the lower the pressure, the higher the score of PSS-14, the higher the pressure; therefore, scale scores were negatively correlated. The SNS-14-CHI was found to have Cronbach’s > 0.8 for both the overall scale and subscales, retest reliability > 0.7, and split-half reliability > 0.8 in the Chinese undergraduate nursing intern population, indicating good reliability of the translation scale [35]. All these results suggest that the SNS-14-CHI is relatively stable, and all indicators are within a reasonable range, which can be used as a reliable evaluation tool to assess the stress of Chinese nursing students.

Differences in stress between different household economies. The results showed that there was a significant difference (*p* < 0.05) in the stress levels of participants with different monthly family incomes, with those with low monthly family incomes showing high-stress levels. The family economic level is a factor that influences nursing interns’ stress [14], probably due to the fact that undergraduate nursing interns with a high monthly family income are able to receive more family support in terms of finances.LouJH et al. [36]. also showed that an increase in family support reduces stress in life. Therefore, participants with low family financial levels were more likely to experience higher levels of stress.

There was a significant difference in participant stress in terms of whether they liked the nursing profession or not. The results showed that participants who preferred nursing careers had lower levels of stress than those who did not prefer nursing careers, which is consistent with the findings of previous studies [37]. Similarly, Hamaideh et al. [38] reported that whether or not they liked the nursing profession was an influencing factor on students’ stress. It was found that students who liked the nursing profession tended to have a stronger sense of professional identity [39]. Nursing students with a stronger sense of professional identity may be more motivated to learn and adapt to the clinical environment more quickly, and as a result, they have lower levels of stress during their clinical placements [40]. Therefore, all of the above studies proved that they had lower stress levels compared to students who disliked the nursing profession.

## Limitations

There are some limitations in this study. First, although the sample size of this study met the criteria, the sample was selected in a concentrated manner, the survey respondents were only undergraduate nursing interns in Liaoning Province, and women were higher than men, which was not representative of China, and a multicenter, extensive sample survey should be conducted in the future to verify the adaptability of the instrument further. Second, the respondents’ questionnaire results were self-reported, and bias in the study report is inevitable. Although the fitting results of this study have passed the CFA, the discriminant and convergent validity between the information structures need to be further verified in future studies.

## Conclusion

This study was the first to examine the cross-cultural Validity of the SNS-14-CHI and was shown to have good psychometric properties in a population of Chinese undergraduate nursing interns. In contrast, the SNS-14-CHI was shown to have good reliability and validity.

### Electronic supplementary material

Below is the link to the electronic supplementary material.

Supplementary Material 1

## Data Availability

The datasets used and/or analysed during the current study are available from the corresponding author on reasonable request.
