# Supplementary material for: Psychometric evaluation of the student nurse stressor-14 scale for undergraduate nursing interns
Source: BMC Nurs. 2023 Dec 8;22:468. doi: 10.1186/s12912-023-01631-z (PMC10704624; doi:10.1186/s12912-023-01631-z)
Supplement: Supplementary file 1 — Supplementary Material 1 [file 12912_2023_1631_MOESM1_ESM.docx]

**The English version of the scale is provided below.**

**We are interested in examining levels of stress on student clinical placements. For each item below, please place a circle around the number which best represents your level of stress:**

| Stressor | Highly  Stressed | Stressed | Neutral | Moderately  Stressed | Not  Stressed |
| --- | --- | --- | --- | --- | --- |
| 1.preceptor relationships | 1 | 2 | 3 | 4 | 5 |
| 2.When you learn about the treatment of staff | 1 | 2 | 3 | 4 | 5 |
| 3.Clinical skills(When using currently acquired clinical skills to solve problems for patients) | 1 | 2 | 3 | 4 | 5 |
| 4.Feelings about acquiring knowledge about different medications (pills, injections, etc.) | 1 | 2 | 3 | 4 | 5 |
| 5.Being prepared/while undertaking the clinical placements | 1 | 2 | 3 | 4 | 5 |
| 6.Placement workload | 1 | 2 | 3 | 4 | 5 |
| 7.Academic workload | 1 | 2 | 3 | 4 | 5 |
| 9.Staffing levels | 1 | 2 | 3 | 4 | 5 |
| 10.clinical nursing teachers relationships | 1 | 2 | 3 | 4 | 5 |
| 11.Patient relationships | 1 | 2 | 3 | 4 | 5 |
| 12.Number of work days per week | 1 | 2 | 3 | 4 | 5 |
| 13.Facilities e.g.canteen | 1 | 2 | 3 | 4 | 5 |
| 14.Missing days on placement(When you are absent from work) | 1 | 2 | 3 | 4 | 5 |
| 15.Length of journey to placement (Internship hospital to accommocation) | 1 | 2 | 3 | 4 | 5 |
